# Addition of Everolimus Post VEGFR Inhibition Treatment Failure in Advanced Sarcoma Patients Who Previously Benefited from VEGFR Inhibition: A Case Series

**DOI:** 10.1371/journal.pone.0156985

**Published:** 2016-06-13

**Authors:** Adam C. ElNaggar, John L. Hays, James L. Chen

**Affiliations:** 1 Division of Gynecologic Oncology, Department of Obstetrics and Gynecology, The Ohio State University, Columbus, Ohio, United States of America; 2 Division of Medical Oncology, Department of Internal Medicine, The Ohio State University, Columbus, Ohio, United States of America; 3 Division of Bioinformatics, Department of Biomedical Informatics, The Ohio State University, Columbus, Ohio, United States of America; Johns Hopkins University, UNITED STATES

## Abstract

**Background:**

Patients with metastatic sarcoma who progress on vascular endothelial growth factor receptor inhibitors (VEGFRi) have limited treatment options. Upregulation of the mTOR pathway has been demonstrated to be a means of resistance to targeted VEGFRi in metastatic sarcoma.

**Patients and methods:**

Retrospective cohort study to evaluate the clinical benefit at four months of combining mTOR inhibition (mTORi) via everolimus with VEGFRi in patients who have derived benefit from single-agent VEGFRi but have progressed. Patients with recurrent, metastatic soft tissue or bone sarcomas who progressed after deriving clinical benefit to VEGFRi beyond 12 weeks were continued on VEGFRi with the addition of everolimus (5 mg daily). Progression free survival was measured from start of VEGFRi to disease progression on single agent VEGFRi as well as from the addition of everolimus therapy to disease progression or drug discontinuation due to toxicity. Clinical benefit was defined as stable disease or partial response at 4 months.

**Results:**

Nine patients were evaluated. Two patients did not tolerate therapy due to GI toxicity and one elected to discontinue therapy. Of the remaining six patients, the clinical benefit rate at four months was 50%. Progression free survival (PFS) for these patients was 3.1 months ranging from 0.5 to 7.2 months with one patient remaining on combination therapy.

**Conclusion:**

In this heavily pre-treated, advanced sarcoma population, the addition of mTOR inhibition to VEGFRi based therapy resulted in a clinical benefit for a subset of patients. Prospective studies will be needed to verify these results.

## Introduction

Soft tissue and bone sarcomas account for less than 1% of all adult cancers [1]. While advancements in therapy have been made, median survival after development of distant metastases is 11 to 15 months [2]. Multiagent cytotoxic regimens have demonstrated response rates ranging from 16 to 46% in this population [3,4], however tolerability remains a concern.

Sarcomas, as with many other tumors, require the recruitment of circulating endothelial progenitor cells to initiate and sustain new blood vessels from preexisting vessels[5], making the vascular endothelial growth factor receptor (VEGFR) a key target for therapy. Targeted therapies, particularly against VEGFR, have become a useful addition to our therapeutic armament as demonstrated by the vascular endothelial growth factor receptor inhibitor (VEGFRi) pazopanib receiving FDA approval [6], and new, similarly promising, phase III data for regorafenib in the REGOSARC trial [7]. Additionally, other VEGFRi’s, including sorafenib and sunitinib have also demonstrated activity in soft tissue or bone sarcomas with progression free survival on the order of 4 months [8,9]; similar to that seen in both the REGOSARC and PALLETTE trials [6,7].

Although tumor angiogenesis activity is initially decreased with VEGFR inhibition, the development of resistance may be mediated by an upregulation of the phosphoinositide-3 kinase (PI3K)/Akt/mammalian target of rapamycin (mTOR) pathway [10–12]. Trials with single agent mTOR inhibition have provided clinical benefit at 16 weeks on the order of 13 to 27% in metastatic soft tissue and bone sarcomas[13,14]. While the single agent activity of TORC1 inhibitors is somewhat limited in STS, they may still have a role in mediating *acquired resistance* to VEGFRi. TORC1 activation has been demonstrated in preclinical models to be an escape mechanism for the development of resistance to anti-angiogenesis treatment [10]. The addition of clinically available mTOR inhibitors (temsirolimus, everolimus, and ridaforolimus) to an angiogenesis inhibitor may be a useful approach in extending the proven activity of VEGFR inhibition in patients with soft tissue or bone sarcomas that have previously responded to VEGFR inhibition. Phase I and II trials evaluating the combination of angiogenesis and mTOR inhibition in patients with refractory solid tumors [15], osteosarcoma [16], and metastatic clear cell renal cancer [17] have demonstrated tolerability and clinical benefit at 6 months on the order of 27 to 45%. In this case series, we sought to evaluate the value of adding everolimus after progression on single agent VEGFRi to patients with soft tissue or bone sarcomas who received clinical benefit from VEGFRi. This study will provide evidence to support the hypothesis that the addition of mTOR inhibition may overcome acquired resistance to VEGFRi in those patients with an initial favorable response to VEGFRi.

## Methods

### Patient selection

**Fig 1:**
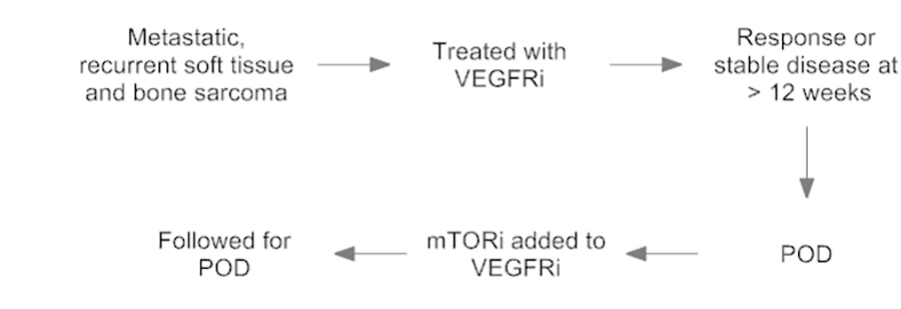
Patient flow chart on VEGFRi & VEGFRi + mTORi. VEGFRi = vascular endothelial growth factor receptor inhibitor; POD = progression of disease; mTORi = mammalian target of rapamycin inhibitor

After approval from The Ohio State Institutional Review Board (OSU:2014E0450), we conducted a retrospective, observational study on patients diagnosed with soft tissue or bone sarcomas between 2008 and 2015 who were treated at The Ohio State University Comprehensive Cancer Center. Patients were eligible if they had received single agent VEGFRi (pazopanib, sunitinib, or sorafenib) in the recurrent setting and achieved clinical benefit at 12 weeks. Twelve weeks was chosen as patients on PALETTE trial who received placebo had a median PFS of 1.6 months. We wanted to ensure that patients who were receiving TKI were likely to be benefitting and therefore chose a conservative cutoff of roughly twice the placebo PFS. Patients also must have and received the addition of everolimus at progression (Fig 1). Everolimus was chosen given our familiarity with the agent in treating other tumor types, its oral formulation, and prior dose finding studies in combination with pazopanib and sorafenib[15,16]. Data regarding age, sex, histology, stage at presentation, prior treatments, and survival were collected.

### Efficacy evaluation

We defined clinical benefit of treatment as stable disease or tumor shrinkage at four months after the addition of everolimus to VEGFRi. Four months was chosen as single agent everolimus has been previously reported to be 1.9 months [14]. We conservatively chose twice the PFS to reduce the number of false positive responses. Response to therapy was classified by complete response, partial response, stable disease, and progressive disease according to RECIST 1.1 [18]and PERCIST 1.0 [19]. Clinical benefit was defined as a complete response, partial response or stable disease. Imaging was generally scheduled to be performed at 6-week intervals unless clinical progression or symptoms necessitated earlier imaging studies.

### Statistical analysis

We calculated progression-free survival from the date of the first administration of VEGFRi/mTORi combination until the date of objective disease progression or death from any cause. All confidence intervals that are reported are two-sided. All analyses were performed in JMP®, Version 11.0, SAS Institute Inc., Cary, NC, 1989–2007.

## Results

### Patient characteristics

Of 48 patients treated with VEGFRi during the study time frame, nine patients were identified as meeting all inclusion criteria. Histologic types and patient characteristics are listed in Table 1. The median age was 48 (range 29–61) and the median number of prior therapies was 3 (range 1–3). Seven patients received pazopanib (400-800mg daily), while one patient each received sunitinib (37.5mg daily) and sorafenib (400mg BID). Median PFS for single agent VEGFRi was 8.3 months (range 3.3–10). At the time of progression, all patients were continued on VEGFR inhibition and received the addition of everolimus (5 mg daily). In combination with everolimus, pazopanib was dosed at 400mg, sunitinib at 37.5mg, and sorafenib at 200mg BID. All patients were evaluated with computed tomography (CT) scans every 8 weeks or if clinically indicated (median 4.1 weeks, range 2.1–10.4 weeks). Five patients also received FDG positron emission tomography (PET) imaging per treating physician preference. Two patients received adjuvant external beam radiotherapy and two patients received palliative external beam radiation >6 weeks prior to going on TKI therapy.

**Table 1:** Patient characteristics and treatments.

Patient	Age	Sex	Histology	Front-line therapy	VEGF inhibition
1	44	F	Non-uterine leiomyosarcoma	Adriamycin	Pazopanib 400 mg
2	29	M	Metastatic alveolar soft part sarcoma	Adriamycin/ifosfamide/mesna	Sunitinib 37.5 mg
3#	46	F	Osteogenic sarcoma (small cell variant)	Cisplatin/Adriamycin	Sorafenib 400 mg
4#	45	F	Non-uterine leiomyosarcoma	Adriamycin/ifosfamide/mesna	Pazopanib 400 mg
5*	61	M	Ewings variant sarcoma	VAC/IE	Pazopanib 400 mg
6	58	F	Desmoplastic small round cell tumor	VAC/IE	Pazopanib 400 mg
7	48	F	Non-uterine leiomyosarcoma	Gemcitabine/paclitaxel ± investigational agent^ⱡ^	Pazopanib 400 mg
8	55	F	Non-uterine leiomyosarcoma	Gemcitabine/paclitaxel ± investigational agent^ⱡ^	Pazopanib 600 mg
9#	55	F	Dedifferentiated chondrosarcoma	Pazopanib	Pazopanib 400 mg

VAC/IE = vincristine, adriamycin, cyclophosphamide alternating with ifosfamide and etoposide

*Patient still on therapy.

#Discontinued therapy secondary to intolerability

ⱡ Ontuxizumab (MORAb-004)

**Fig 2:**
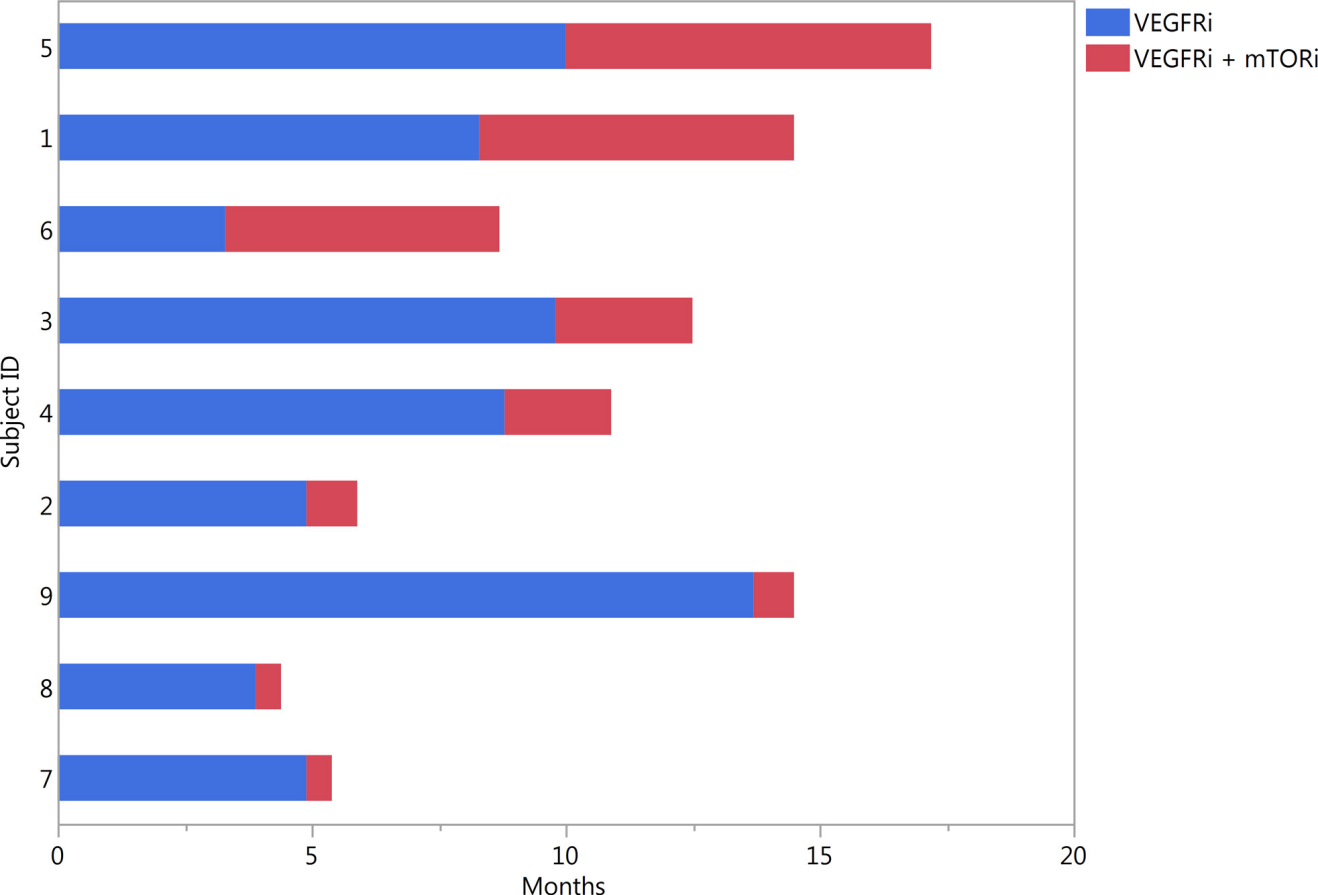
Stacked bar chart of response duration to VEGFRi & VEGFRi + mTORi. VEGFRi = vascular endothelial growth factor receptor inhibitor; mTORi = mammalian target of rapamycin inhibitor Subject 5 remains on pazopanib and everolimus therapy

VEGFRi with everolimus resulted in an initial disease stability for a majority of patients. At initial imaging with CT, 7 patients demonstrated stable disease and 2 patients had progression of measurable disease (S1 Table). Although there were no partial responses by RECIST, one patient had an 11% reduction in tumor volume by RECIST criteria and a corresponding partial response by PERCIST criteria. Duration of clinical benefit to VEGFRi monotherapy and VEGFRi/mTORi can be seen in Fig 2. Three patients discontinued therapy prior to progression: two due to grade 3 nausea and vomiting and one elected to discontinue systemic therapy entirely.

Three patients derived meaningful clinical benefit with progression-free intervals of 6.2, 7.2, and 5.4 months. At the time of writing, 7 patients remain alive with disease. Patient 5, who has a EWS/NFATc2 type Ewing's variant sarcoma, remains on pazopanib and everolimus therapy. Two patients have died of disease. All patients who achieved a meaningful benefit received pazopanib along with everolimus. Of the remaining 3 patients who did not receive clinical benefit, 1 died within 8 weeks of discontinuing therapy and 2 patients remain alive with disease at 2 and 6 months.

## Discussion

Recurrent, metastatic sarcomas are difficult to treat and are often resistant to standard cytotoxic chemotherapy. Targeted therapies, such as VEGFRi, are emerging and showing promise in this setting; however these responses are often short lived. To improve the duration of responses, it is crucial to understand and target possible pathways that lead to primary and acquired resistance to VEGFRi therapy. In preclinical and clinical studies, targeting mTOR has proven to be a novel method to inhibit cells that have acquired resistance to angiogenesis inhibition [12,20–23]. Here we provide the first multi-patient clinical evidence that the mTOR inhibitor everolimus may play a role in overcoming acquired resistance to a VEFGRi in select patients with metastatic, recurrent STS.

Patients in this case series had a median VEGFRi duration of 35.7 weeks (range 14–58.6 weeks). This is a very favorable subgroup on VEGFRi as compared to the median treatment duration of 16.4 weeks seen in the PALETTE trial [6]. We selected this population to address the question of acquired resistance to VEGFRi in STS and not the separate, but equally important, question of primary resistance to VEGFRi. The addition of everolimus resulted in clinical benefit at 4 months for 3 of 6 patients who we were able to assess for progression. Comparatively, everolimus monotherapy, based on phase II studies of patients with soft tissue or bone sarcomas, demonstrated a clinical benefit of 13 to 27% at 4 months [13,14].

In the current study, patients were not selected for PI3K/AKT/mTOR pathway alterations and no molecular information is available. A recent phase I trial of pazopanib/everolimus demonstrated that the presence of molecular alterations in the PI3K/AKT/mTOR pathway (PTEN loss, PIK3CA mutations, AKT mutation, NF1 mutation, or RICTOR amplification) did not predict response in patients with advanced solid tumors refractory to standard therapy (p = 0.764) [15].

Even though all patients who achieved meaningful benefit were on the combination of pazopanib and everolimus, other VEGFRi’s were used in this series. The small sample size limits evaluation of statistical significance of this observation. Pazopanib is the only TKI FDA approved for the treatment of soft tissue sarcomas and has been demonstrated to be well tolerated when combined with mTOR inhibition, including sequential addition for the reversal of TKI resistance in a case report [21]. The addition of everolimus in patients with acquired resistance to VEGFRi resulted in 3/6 evaluable patients receiving clinical benefit at 4 months in our small series of patients with recurrent and metastatic sarcoma. Our case series lends support for further prospective exploration in the context of a clinical trial of combining mTOR inhibition with VEGFR inhibition in this difficult to treat patient population.

## Supporting Information

S1 Table Initial response to VEGFRi + mTORi therapy post VEGFRi failure and timing of initial evaluation. * Patient still on therapy. # Progression of disease has defined by new lesions. **Patients scanned at earlier than 6 weeks post initiation of therapy were evaluated secondary to either clinical progression or tolerability of the combination.
(DOCX)
