# Supplementary material for: Addition of Everolimus Post VEGFR Inhibition Treatment Failure in Advanced Sarcoma Patients Who Previously Benefited from VEGFR Inhibition: A Case Series
Source: PLoS One. 2016 Jun 13;11(6):e0156985. doi: 10.1371/journal.pone.0156985 (PMC4905654; doi:10.1371/journal.pone.0156985)
Supplement: S1 Table — * Patient still on therapy. # Progression of disease has defined by new lesions. **Patients scanned at earlier than 6 weeks post initiation of therapy were evaluated secondary to either clinical progression or tolerability of the combination. (DOCX) [file pone.0156985.s001.docx]

Supplemental Table 1: Initial response to VEGFRi + mTORi therapy post VEGFRi failure and timing of initial evaluation.

| Pt ID | % Change from baseline | Initial Response | Initial Evaluation Timepoint (months) |
| --- | --- | --- | --- |
| 5* | -11.6 | SD | 1.9 |
| 3 | -3.3 | SD | 2.6 |
| 4 | -0.7 | SD | 1.0** |
| 6 | 0.8 | SD | 1.7 |
| 1 | 5.6 | SD | 2.4 |
| 9 | 11.7 | POD^#^ | 0.9** |
| 2 | 14.1 | SD | 1.0** |
| 8 | 14.4 | SD | 0.5** |
| 7 | 30.9 | POD | 0.6** |

* Patient still on therapy.

# Progression of disease has defined by new lesions.

**Patients scanned at earlier than 6 weeks post initiation of therapy were evaluated secondary to either clinical progression or tolerability of the combination
